# Syndecan-3 in Inflammation and Angiogenesis

**DOI:** 10.3389/fimmu.2019.03031

**Published:** 2020-01-09

**Authors:** Samantha Arokiasamy, Michaela J. M. Balderstone, Giulia De Rossi, James R. Whiteford

**Affiliations:** ^1^Barts and the London School of Medicine and Dentistry, William Harvey Research Institute, Queen Mary University of London, London, United Kingdom; ^2^Department of Cell Biology, UCL Institute of Ophthalmology, London, United Kingdom

**Keywords:** heparan sulfate, syndecan, HIV, rheumatoid arthritis, angiogenesis

## Abstract

Syndecans are a four member multifunctional family of cell surface molecules with diverse biological roles. Syndecan-3 (SDC3) is the largest of these, but in comparison to the other family members relatively little is known about this molecule. SDC3 null mice grow and develop normally, all be it with subtle anatomical phenotypes in the brain. Roles for this molecule in both neuronal and brain tissue have been identified, and is associated with altered satiety responses. Recent studies suggest that SDC3 expression is not restricted to neuronal tissues and has important roles in inflammatory disorders such as rheumatoid arthritis, disease associated processes such as angiogenesis and in the facilitation of infection of dendritic cells by HIV. The purpose of this review article is to explore these new biological insights into SDC3 functions in inflammatory disease.

## Introduction

The proteoglycans are a diverse family of molecules with multiple roles in development, health and disease (1). Heparan Sulfate Proteoglycans (HSPGs) form a subset of these, and these can be secreted extracellular matrix molecules or membrane associated (1). The principal families of membrane associated HSPGs are the glypicans and syndecans. Glypicans are distinguished from syndecans by the fact they are tethered to the cell membrane via glycosylphosphatidylinositol anchors. Syndecans (SDC) are a four member family of type 1 transmembrane proteins consisting of two related sub-families based on sequence homology. SDC2 and 4 form one family and SDC1 and 3 form the other. All syndecans have a short highly conserved cytoplasmic domain, a single transmembrane domain, and a larger extracellular core protein. Syndecan ectodomains are far less conserved between family members or species. However, they do possess Ser-Gly GAG attachment motifs usually surrounded by acidic amino acid residues. Every cell type expresses at least one syndecan, and roles in cell adhesion, migration, growth factor signaling, receptor trafficking and ion channel modulation have been identified [for reviews see (1–5)]. Of the four family members, perhaps the least well-understood is Syndecan-3 (SDC3). Predominantly, reported roles for this molecule have been exclusively related to the brain and nervous system, but recent studies are revealing roles for SDC3 in other important biological processes such as inflammation and angiogenesis. This review aims to explore this work and provide novel insight into the biology of SDC3.

## Syndecan-3 Molecular Architecture

**Figure 1:**
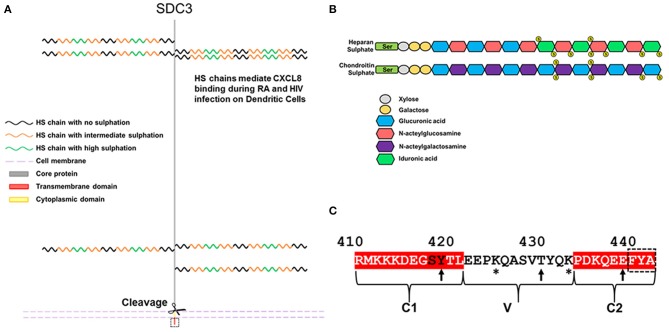
Schematic diagram and features of SDC3. **(A)** Human SDC3 is 442aa in length and contains seven potential sites for GAG substitution. Most functions ascribed to SDC3 relate to interactions through its HS chains. HS chains are heterogenous in nature and contain regions of low, intermediate and high sulphation. **(B)** Structure of HS and CS. **(C)** The cytoplasmic domain of SDC3 is 33 amino acids in length and shares conserved C1 and C2 domains with other syndecan family members. The C-terminal PDZ binding domain is indicated by a dashed black box. Potential sites of tyrosine phosphorylation are indicated by arrows and lysine residues potentially involved in phosphoinositide binding are indicated by asterisks. The conserved SY in the C1 domain, which can be phosphorylated in other syndecan family members are denoted by black letters.

The syndecan-3 (N-syndecan) gene was first cloned from rat Schwann cells and was the third syndecan of the four member family to be identified (6, 7). It bears significant homology to syndecan-1, although unlike syndecan-1, it has a homolog in fish (8). Of the four family members, syndecan-3 (SDC3) is the largest, consisting of 442 amino acids in humans. In mammals, the SDC3 ectodomain contains seven potential glycosaminoglycan (GAG) attachment sites. Four are situated toward the N-terminus and the remaining three reside closer to the transmembrane domain (Figure 1A). The type and extent of GAG substitution on syndecan-3 is likely to be cell type and tissue dependent. Experiments using heparan sulfate (HS) and chondroitin sulfate (CS) degrading enzymes would suggest that SDC3 (at least when purified from brain tissue) possesses both HS and CS chains (9).

HS chains consist of repeated disaccharide units consisting of glucuronic acid and N-acetylglucosamine and chain lengths can range from 50 to 200 disaccharides. It is synthesized via a stepwise series of enzymatic reactions involving multiple transferases; briefly N-acetyglucosamine within a chain can undergo N-sulphation followed by the epimerisation of some glucuronic acids, this is followed by the sequential addition of first 2-O, 6-O, and 3-O sulfate groups. The complexity of this process means that HS chains are heterogenous in terms of sulphation and there is considerable evidence of HS chains having domains of high, intermediate and low sulphation [for review see (10), Figure 1B. CS is structurally distinct to HS on the basis that it is comprised of repeating disaccharide units consisting of glucuronic acid and n-acetylgalactosamine (Figure 1B). CS chains can also be sulphated although the extent and heterogeneity of this is not as great as HS. The majority of reported interactions between SDC3 and biologically active molecules are with its HS chains, and far less is understood as to the role or function of SDC3 CS chains. The extracellular core protein also possesses a domain rich in threonine and proline residues, which is characteristic of mucin-like proteins, and are potential sites for *O-*linked glycosylation (6).

Syndecan shedding is a regulated process whereby some or all of the extracellular domain of the molecule is cleaved from the cell surface by the action of matrix-metalloproteases (MMPs). Shed SDC3 has been observed in conditioned media in a variety of experimental systems, although the precise cleavage sites and proteases have yet to be confirmed (11). Predictive algorithms would suggest roles for MMP2, 9, and 14 at multiple cleavage sites but these have not been validated experimentally (3). SDC3, along with the other family members, form SDS insoluble dimers and this self-association is mediated both by residues in the transmembrane domain, and a membrane proximal ERKE motif (12).

The syndecan-3 cytoplasmic domain consists of 33 aa and in common with the rest of the family possesses two conserved domains (C1 and C2) flanking a variable sequence (V region), which is unique to each family member (Figure 1C). Syndecans all have the capability of interaction with PDZ proteins possessing a conserved EFYA motif at the C-terminus of the C2 region. Interactions between SDC3 and PDZ proteins such as syntenin, CASK, TIAM1, synectin, and synbindin have been reported. The SY sequence (Ser^418^, Tyr^419^ in human SDC3, Figure 1C) in the C1 region of SDC3 is common to all syndecans. Studies with SDC4 have shown that phosphorylation of either residue is important for its functionality. Syndecans have proven remarkably recalcitrant to structural analysis, none more so than SDC3, for which there are no structural records in the databases (2). However, structural analysis of peptides corresponding to the syndecan-4 cytoplasmic domain in which this serine residue is phosphorylated show that this alters the conformation of this molecule considerably, and expression of phospho-mimetic mutants of this residue leads to enhanced cell migration (13). In other work, the Y (Tyr^180^ in human SDC4) was identified as a substrate for Src kinase and this has implications for the trafficking of integrins. It would not be surprising if phosphorylation of these conserved residues impacted on SDC3 functionality in a similar fashion (14). Additionally, *in vitro* studies have indicated that the other 2 tyrosines could potentially be phosphorylated, however no functional role for this has been demonstrated (15). The V region of SDC3 also bears similarities to other syndecans in that there are 2 lysine residues (Lys^425^ and Lys^433^). Studies with syndecan-4 have shown the importance of these lysines for binding phospho-inositides (PIP3) and this not only stabilizes the structure of SDC4 cytoplasmic domain but this interaction is also associated with upregulating the activity of PKCα which promotes many of the downstream cell adhesion pathways associated with SDC4 (16, 17). It is therefore also conceivable that the interactions with the SDC3 V region and phospho-inositides could be important for SDC3 functionality.

## Syndecan-3 in the Brain and Nervous Tissue

The association with SDC3 and cells of a neuronal lineage is well-established and a number of phenotypes associated with both the brain and nervous tissue have been identified (18, 19). Although no gross abnormalities are observed in the developing mouse brain, stereological analysis of sections revealed differences in the cellular density of key areas. Specifically, SDC3 null animals have a higher density of cells in deep cortical areas and a reduced number in the superficial cortical layers. This was found to be due to defects in neural cell migration during development and in particular, an interaction between SDC3 and heparin binding growth associated molecule (HBGAM) (20). Syndecans are known to be key players in cell adhesion and migration, so identifying a migration defect in neural cells associated with SDC3 is entirely consistent.

SDC3 is found in other tissues of the brain, immunostaining of wild-type mice revealed SDC3 to be expressed in the hypothalamus, particularly in the paraventricular nucleus and the lateral hypothalamic area (21). SDC3 expression (uniquely amongst the family members) is upregulated in the hypothalamus in response to food deprivation and the situation is reversible once starved animals are refed. Unsurprisingly, food deprived animals exhibit an abnormal desire for food once the deprivation is ended. However, this response is lacking in animals null for SDC3, suggesting a role for SDC3 in feeding behaviors (21). This is further evidenced by the fact that SDC3 animals are partially resistant to obesity when given a high fat diet due to reduced food intake (22).

Several hypothalamic neuropeptides regulate feeding behaviors; for example, agouti-related protein (AgRP), melanin concentrating hormone (MCH) and neuropeptide Y stimulate increased feeding behaviors, whereas α-melanocyte stimulating hormone (αMSH) and corticotrophin releasing hormone (CRH) serve to inhibit pathways associated with increased feeding (23). It is known that many of the interactions between these peptides and their receptors at least in part have an HS involvement. There is a complex interplay between these neuropeptides and SDC3 is thought to have a role in promoting the antagonism between AgRP and αMSH for the melanocortin receptor MC-4R (24, 25). In essence, when animals are starved, SDC3 is upregulated and this promotes the binding of AgRP to MC-4R leading to a reduction in anti-satiety signals, and enhanced feeding. In animals null for SDC3, this interaction does not occur meaning αMSH is free to bind MC-4R leading to a reduction in the desire to feed in these animals. Interestingly, another consequence of starving is an upregulation of Tissue Inhibitor Metalloprotease-3 (TIMP3), this acts as an inhibitor to a variety of MMPs which also have key roles in syndecan shedding. This provides a means of regulating feeding behaviors since under conditions of starvation, SDC3 shedding is inhibited thus promoting occupation of MC-4R by AgRP and promoting feeding. On the other hand, under feeding conditions TIMP is down regulated, SDC3 shedding is up regulated and the satiety signal through the αMSH/MC-4R axis is promoted (26, 27). The hypothalamus also has key roles in modulating reward processing in addictive behaviors and SDC3 also has a role to play in these. Cocaine administration increases SDC3 expression in the lateral hypothalamic area and SDC3 knockout mice actually exhibit more addictive behaviors than wild-type counterparts. SDC3 null animals were more susceptible to cocaine addiction, a situation that could be reversed upon re-expression of SDC3. Glial cell line-derived growth factor (GDNF) acts to both increase and decrease cocaine self-administration behaviors in rodents through its interactions with a signaling complex consisting of the receptor GFR-α1 and the tyrosine kinase c-Ret (28). SDC3 is thought to disrupt this signaling complex since it can also bind GDNF and as such, is a potentially important target for treating addictions.

## Syndecan-3 in Disease

It is very evident from the studies described above that SDC3 plays a critical role in both brain development and behavior. However, other roles for SDC3 on endothelial cells (ECs) and leukocytes are emerging in inflammatory responses. The purpose of the remainder of this review is to examine the roles of SDC3 in the context of inflammatory disease (rheumatoid arthritis), angiogenesis and also HIV infection.

### Syndecan-3 in Rheumatoid Arthritis

SDC3 is expressed on endothelia in both rheumatoid and non-rheumatoid synovia and is thought to have roles in binding chemokines, specifically CXCL8, during the progression of the disease (29, 30). Interestingly, mice null for SDC3 are protected in a model of antigen-induced arthritis (methylated BSA induced), in which clinical scores, leukocyte recruitment and cartilage damage were all significantly less than wild-type animals (31). This is a common feature of syndecan null animals, in that phenotypes only emerge when the animals are challenged and more often than not, deletion of syndecans leads to less severe disease progression, suggesting a role only in pathological scenarios. This study also highlighted a slight paradox; in models of both dermal and cremasteric inflammation, leukocyte rolling and adhesion was elevated in SDC3-null mice, suggesting an anti-inflammatory role for this molecule (31). This therefore suggests distinct roles for SDC3 depending on the vascular bed. SDC3 is thought to facilitate chemokine interactions via binding to its GAG chains, and it is likely that the extent and sulphation pattern of the HS chains in ECs from different tissues will vary. Administration of recombinant forms of SDC3 ectodomains, expressed in mammalian cells, have also been used in both collagen- and methylated BSA-induced murine inflammatory arthritis models with efficacious effects indicating both the therapeutic potential of targeting this molecule but also the importance of shed SDC3 in disease progression (32).

### Syndecan-3 in Neovascular Diseases

Angiogenesis, the formation of new blood vessels from existing vasculature, and inflammation are intrinsically linked. SDC3-null mice exhibit no gross abnormalities suggesting that the formation of the vasculature in these animals is normal. However, in-depth studies on parameters such as vessel density, vessel diameter, vessel frequency and pericyte coverage have yet to be undertaken. SDC3 is expressed on ECs in early retinal development in rats (33), and has been found expressed on ECs from a variety of tissues. For example, along with SDC4, SDC3 is abundant on cultured human umbilical vein ECs (34), and human coronary artery endothelial cells as well as human coronary artery smooth muscle cells (35). Vascular endothelial growth factor A (VEGFA), alongside a number of other proangiogenic factors are known to bind HS (36, 37), so it would be tempting to propose a role for SDC3 in this context. Interestingly, sequences within the SDC3 extracellular core protein exert anti-angiogenic affects by blocking EC migration (38). A fusion protein consisting of GST fused to the N-terminus of the SDC3 ectodomain inhibits angiogenic sprout formation from aortic explants, and also inhibited EC micro-capillary formation and EC migration. Importantly, this protein was generated in a prokaryotic expression system so lacked any GAG substitutions, indicating that this is an intrinsic property of the core protein (38). This is also in common with other syndecan family members, whose core proteins also exhibit biological activity (39). SDC1, which is closely related to SDC3, has anti-angiogenic sequences within its core protein but they bear no homology to the SDC3 sequence suggesting that the anti-angiogenic properties of the latter may work via a distinct mechanism (40, 41). It remains unclear how these sequences affect the biology of the full length molecule—do they bind receptors *in cis*, as is the case for SDC1 (41) or *in trans* when shed as is the case for SDC2 (42)?

**Figure 2:**
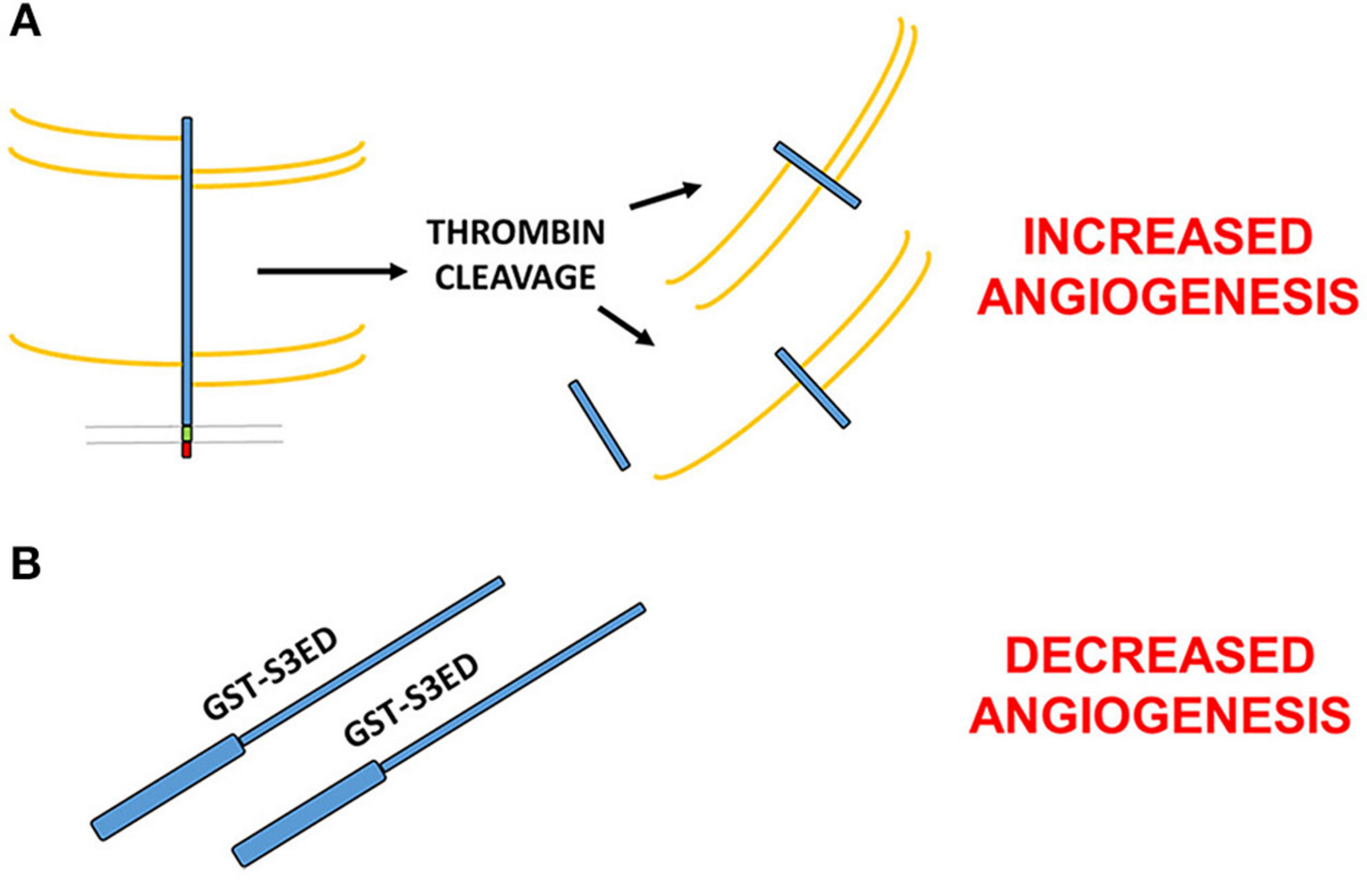
Contrasting roles for the SDC3 ectodomain in angiogenesis. **(A)** Thrombin cleaved fragments of the SDC3 ectodomain promote pro-angiogenic processes such as EC junctional disassembly (43). **(B)** Full length bacterially expressed SDC3 extracellular core protein fused at the N-terminus to GST inhibits angiogenesis in a number of *in vitro* and *ex vivo* models (38). Since the thrombin cleavage sites of SDC3 are not known, it is possible that one or more of the cleavage products is pro-angiogenic whereas the full length sequence acts to inhibit new blood vessel formation.

A key step in the formation of new blood vessels, and indeed inflammation, is the disassembly of EC junctions, enabling both the migration and proliferation of ECs and vascular leakage during disease. Thrombin-cleaved fragments of the SDC3 (and SDC4) ectodomain have been shown to promote this process in the human lung microvasculature, which has ramifications for conditions such as sepsis or thrombotic disease states, where thrombin is activated (43). This would suggest opposing roles for the SDC3 ectodomain in angiogenesis; on the one hand, studies would suggest that the core protein independent of GAG chains can inhibit the process (44), whereas fragments of SDC3 cleaved from mammalian cells with their GAGs intact appear to promote vascular permeability and EC migration. It is conceivable that since the thrombin cleavage sites in the SDC3 extracellular core proteins are not known, smaller fragments of the mature protein may in fact exhibit pro-angiogenic effects (Figure 2).

### Syndecan-3 Interactions With Human Immunodeficiency Virus 1 (HIV-1)

Dendritic cells (DCs) are antigen presenting cells and play a critical role in identifying and capturing pathogens in peripheral tissues and subsequently priming T cells in the lymph nodes to initiate adaptive immune responses. Sexual transmission is the main route of HIV-1 dissemination and in the absence of surface lesions, the genital epithelia presents a barrier to viral crossing (45, 46). HIV circumvents this by using DCs as molecular Trojan horses to cross this normally impenetrable epithelium (47). DCs induce virus-specific CD8+ T cell responses by presenting antigens bound by major histocompatibility complex molecules class-I (MHC-I) to these T cells, thus activating them. DCs infected with viruses can use viral proteins which are endogenously synthesized from viral replication as antigens for presentation on MHC-I in a process commonly called direct presentation (48). On the other hand, DCs not infected with viruses must engulf exogenous viral antigens for presentation to CD8+ T cells via a process known as cross-presentation (49). DCs have been shown to be susceptible to HIV-1 infection (50). Therefore, direct presentation usually takes place in the context of normal HIV transmission.

A number of cell surface receptors have been implicated in facilitating both the recognition, binding and transmission of the HIV-1 virus; different receptors have been shown to interact with HIV-1 on DCs, such as the C-type lectin DC-SIGN (51, 52), the mannose receptor (53), langerin (54), and CD4 (53). Most of these studies were carried out using HIV-1 glycoprotein gp120 but other experiments using HIV-1 particles suggest that other unknown receptors are also involved (55–57).

DC-SIGN, which binds with high affinity to ICAM-3 present on resting T cells, was discovered to play a key-role in the dissemination of HIV-1 by DCs (51, 58). It does not function as a receptor for HIV-1 virus entry into DCs but instead promotes efficient capture of HIV-1 viruses in the periphery and facilitate their transport to secondary lymphoid tissues to enhance infection *in trans* of cells that express CD4 and chemokine receptors (i.e., T cells) (51). More recently, de Witte et al. (59) found SDC3 to be highly expressed by DCs, namely immature monocyte-derived DCs, and was identified as a major specific HIV-1 attachment receptor. SDC3 captures HIV-1 through interaction with its HS chains and the viral envelope protein gp120; it also acts to stabilize the captured virus, enhance DC infection and like DC-SIGN, promotes HIV transmission to T cells. The authors also found that neutralization of both SDC3 and DC-SIGN leads to the complete impairment of HIV-1 binding to DCs and transmission to T cells. Neutralization of SDC3 alone led to partial inhibition of HIV-1 transmission. As current anti-viral treatments are only aimed at blocking viral replication in T-cells, this opens up a whole new avenue in terms of developing an HIV microbicide that targets DC-SIGN and SDC3 on DCs.

Interestingly, HIV-1 infected individuals known as HIV controllers (HICs) who are able to control viral replication without anti-retroviral therapy exist, but are rare. DCs from these individuals express higher levels of SDC3 and DC-SIGN and have been shown to be less susceptible to HIV-1 infection than cells from healthy donors. On the contrary, DCs from HICs show an enhanced capacity to capture HIV-1 when compared to cells from healthy donors or HIV-1 patients currently on anti-retroviral treatment with suppressed viral load (60). High levels of both SDC3 and DC-SIGN on DCs have previously been confirmed to play crucial roles in facilitating HIV-1 capture and this would, at first, seem contradictory (51, 58, 59). However, the combination of being less permissible to HIV-1 infection and having increased capacity to capture HIV-1 particles may allow DCs from HICs to preserve their function from the deleterious effect of infection—all of which might facilitate the induction of HIV-specific CD8+ T cells by cross-presentation in the context of low viremia.

## Concluding Remarks

In this review, we have explored the diverse roles that the HSPG, SDC3, plays in a range of disease and developmental contexts. Although many phenotypes have been described, there is still a great deal to learn about how SDC3 is actually functioning in these scenarios. SDC1, 2 and 4 have been intrinsically linked with integrins and in particular modulating cell adhesion and migration responses. SDC1 has been shown to modulate the activity of a number of αV integrin heterodimers, as has SDC4 (14, 61). Similarly β1 integrin heterodimers have also been associated with SDC2 and 4 (14, 42, 62). The consequences of these interactions lead to cell migration and adhesion defects when they are compromised. Based on this, it seems likely that SDC3 would also interact and/or modulate integrin activity although further research is required to establish this for sure.

In many instances, it is interactions with SDC3 HS chains that appear to be driving the biological effects observed, whether it be acting to bind neuropeptides, inflammatory mediators or viral particles. However, this does raise the question as to what is driving this selectivity of the molecules toward SDC3 HS. In many cases, the other syndecans and indeed glypican family members are present in abundance but cannot perform the same functions as SDC3 despite possessing HS chains. One possibility is that sequences specific to SDC3 may influence the type and nature of GAG substitution, and it is this that differentiates SDC3 from other HSPGs. This would not be without precedent since studies have shown that sequences in the SDC2 ectodomain can influence the sulphation pattern of SDC2 HS (63). Alternatively, it could be that the expression and localization of SDC3 in cells could be more exquisitely regulated than first thought. Studies in the brain have shown that SDC3 expression can be induced both by starvation and narcotic substances and this is not the case for other family members. SDC3-null animals (like the other family members) develop normally for the most part and it is when challenged that phenotypes are observed. This is suggestive that SDC3 expression is governed by factors associated with these challenges. Molecules such as this make tempting therapeutic targets since it is to be expected that off-target effects would be negligible since the molecule is only having a role in the disease state. It is also entirely possible this selectivity of function of SDC3 HS is actually a combination of both of the above.

The studies described above point to important roles for SDC3 in inflammatory disease which require further investigation and hint that this molecule is important not just in the neuronal context but in other tissues. More detailed studies are required in both the SDC3 null mouse, and patient samples, to explore disease models where inflammation and angiogenesis are a feature. Greater mechanistic insight is required to understand how SDC3 exerts its functions. It remains a possibility that modulating SDC3 function could prove useful in the treatment of the pathologies described above, however, the means by which this could be achieved requires elucidating.

## Author Contributions

JW and SA generated the text and performed the literature searches. MB prepared the figures and GD critically appraised and edited the manuscript.

### Conflict of Interest

The authors declare that the research was conducted in the absence of any commercial or financial relationships that could be construed as a potential conflict of interest.
